# Telomere Length of Circulating Cell-Free DNA and Gastric Cancer in a Chinese Population at High-Risk

**DOI:** 10.3389/fonc.2019.01434

**Published:** 2019-12-17

**Authors:** Yu Shi, Yang Zhang, Lian Zhang, Jun-Ling Ma, Tong Zhou, Zhe-Xuan Li, Wei-Dong Liu, Wen-Qing Li, Da-Jun Deng, Wei-Cheng You, Kai-Feng Pan

**Affiliations:** ^1^Key Laboratory of Carcinogenesis and Translational Research (Ministry of Education/Beijing), Department of Cancer Epidemiology, Peking University Cancer Hospital and Institute, Beijing, China; ^2^Linqu Public Health Bureau, Shandong, China; ^3^Key Laboratory of Carcinogenesis and Translational Research (Ministry of Education/Beijing), Department of Etiology, Peking University Cancer Hospital and Institute, Beijing, China

**Keywords:** cell-free DNA, prospective cohort, gastric cancer, serum, telomere length

## Abstract

**Background:** Telomeres have long been found to be involved in cancer development, while little was known about the dynamic changes of telomere length in carcinogenesis process.

**Methods:** The present study longitudinally investigated telomere alterations of cell-free DNA (cfDNA) in 86 gastric cancer (GC) subjects recruited through a 16-year prospective cohort with 2–4 serums collected before each GC-diagnosis from baseline and three follow-up time-points (a total of 276 samples). As the control, 86 individual-matched cancer-free subjects were enrolled with 276 serums from the matched calendar year.

**Results:** In the 73 pairs of baseline serums from GC and control subjects, shortened telomeres showed increased subsequent GC risk [odds ratio (OR) = 9.17, 95% CI: 2.72–31.25 for 1 unit shortening]. In each baseline gastric lesion category, higher risks of GC progression were also found with shortened cfDNA telomeres; ORs per 1 unit shortening were 6.99 (95% CI: 1.63–30.30) for mild gastric lesions, 6.06 (95% CI: 1.89–19.61) for intestinal metaplasia and 15.63 (95% CI: 1.91–125.00) for dysplasia. With all measurements from baseline and follow-up time-points, shortened telomeres also showed significant association with GC risk (OR = 7.37, 95% CI: 2.06–26.32 for 1 unit shortening). In temporal trend analysis, shortened telomeres were found in GC subjects compared to corresponding controls more than 3 years ahead of GC-diagnosis (most *P* < 0.05), while no significant difference was found between two groups within 3 years approaching to GC-diagnosis.

**Conclusion:** Our findings suggest that telomere shortening may be associated with gastric carcinogenesis, which supports further etiological study and potential biomarker for risk stratification.

## Introduction

Telomeres are tandem repeats of TTAGGG nucleotides at the ends of eukaryotic chromosomes. They have long been shown to maintain chromosome integrity and genomic stability (1, 2). Shortened telomeres can induce cellular senescence or apoptosis (3). Persistent cell division bypassing apoptosis may cause genomic instability and tumorigenesis as a result of shortened telomere in chromosome (4, 5). A recent prospective multi-center cohort study reported that intestinal metaplasia (IM) subjects with shortened telomeres in gastric mucosa were associated with subsequent progression to dysplasia (DYS) or gastric cancer (GC) (6), suggesting that telomere shortening may be involved in the process of gastric carcinogenesis.

The application of tissue biomarkers is restricted for invasive collection and high cost in prevention and clinical practices, especially in a large population setting. Consequently, an increasing number of studies have evaluated telomere length of peripheral blood lymphocytes DNA and GC risk, but reported inconsistent results (7–9). The single time-point measurement in most previous studies has been a major limitation, which made it difficult to investigate the longitudinal alterations of telomere length affected by aging, environmental exposures or carcinogenesis processes.

Circulating cell-free DNA (cfDNA) is becoming a promising target in early detection, therapy response monitor and prognosis evaluation of cancer (10). Serum cfDNA may be released in inflammatory, infection or carcinogenesis processes from a wide spectrum of cells, including necrotic and apoptotic cells, active blood cells and circulating tumor cells. Therefore, studies suggested that the alterations of cfDNA may be more sensitive than peripheral blood leukocyte DNA to reflect the overall organism status for their multiple origins (11–13).

In 1994, we initiated an intervention trial for GC prevention in Linqu County, a rural area in northeast China, which has one of the highest GC mortality and precancerous gastric lesions rates in the world (14, 15). Our previous follow-up study and intervention trial in this area found that *H. pylori* infection, cigarette smoking, and low level of dietary Vitamin C may contribute to the development of GC (16). With multiple serum samples collected from baseline and three subsequent follow-up clinical examinations, this prospective cohort study provided us a unique opportunity to explore the temporal trend and dynamic attrition of cfDNA telomere length during the long-term process of gastric carcinogenesis.

## Methods

### Study Subjects

The details of this intervention trial were described elsewhere (14, 15). Briefly, a total of 3,411 residents aged 35–64 years from 13 randomly selected villages in Linqu County were enrolled in an initial screening program with endoscopic examinations and blood sample collections in 1994. Then, 3,365 eligible subjects were assigned randomly to receive three interventions or corresponding placebos for GC prevention in 1995, including anti-*H. pylori* treatment for 2 weeks, vitamins or garlic supplementations for 7.3 years. To monitor the serum levels of micronutrients after interventions, blood samples were collected from trial participants in 1996 and 1997, respectively. In 1999, an endoscopic screening was performed to follow up the effects of interventions on gastric lesion progression with blood sample collections at the same time. The incidences of cancers were followed from 1995 until 2010, with 106 GC patients identified.

**Figure 1:**
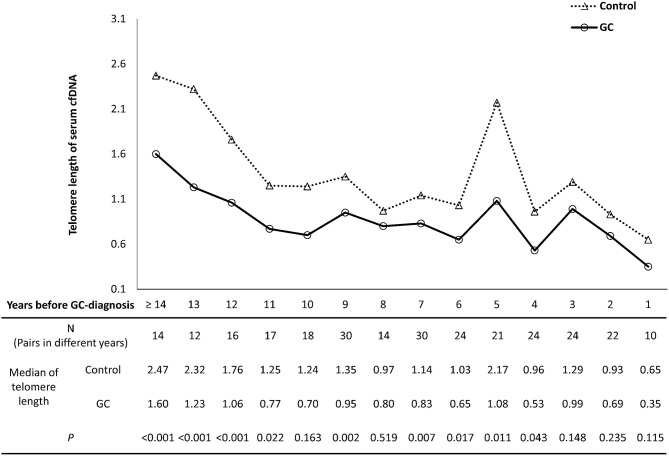
The temporal trends of cfDNA telomere length in GC and control groups. The X axis presents the years before GC-diagnosis of pre-diagnostic samples from GC subjects. The number of sample pairs was shown in each group, and the *P*-values were calculated by linear regression with adjusting age variable between GC and control groups.

For the present study, 86 GC cases were enrolled with 2 to 4 serum samples before each GC-diagnosis from baseline and three follow-up time-points, respectively. Among these GC cases, 79 (91.9%) were pathologically confirmed as 75 (87.2%) intestinal and 4 (4.7%) diffuse type. The locations of the GC were identified in 82 (95.3%) cases, with 54 (62.8%) in angulus or antrum, 20 (23.3%) in body, 6 (6.9%) in cardia, and 2 (2.3%) in pylorus of stomach. A total of 79 (91.9%) GCs had records of lymph node and distant metastasis when initially diagnosed, including 39 (45.3%) with lymph node or distant metastasis, 40 (46.5%) without any kind of metastasis. In all the 86 GC cases, 36 (41.9%) cases had serum samples from all the 4 time-points, 32 (37.2%) cases had samples from 3 time-points, and 18 (20.9%) cases had samples from 2 time-points. A total of 276 pre-diagnostic serum samples were selected, including 73 from baseline, 73 from 1996, 74 from 1997, and 56 from 1999. For each pre-diagnostic sample, the number of years before GC-diagnosis was identified by the time interval between the dates of sample collection and clinical diagnosis, ranging from 1 to 16 years (Figure 1). Since only a small number of samples were collected at 15 or 16 years before GC-diagnosis (*n* = 1 and 5, respectively), they were combined into the group of ≥14 years before GC-diagnosis.

For each GC case, one control without any types of cancer during the follow-up period was randomly selected from the cohort participants, matched by gender, age (< ±5 years) and calendar year of serum sample collection. Finally, a total of 276 serum samples from 86 controls were available for the current study. This study was approved by the Institutional Review Board of Peking University Cancer Hospital.

### Telomere Length Measurement

CfDNA was extracted from 150 μL serum sample using QIAamp DNA Blood Mini kit (Qiagen, CA) according to the manufacturer's protocol and eluted in 40 μL of elution buffer. The telomere length of each DNA sample was determined by quantitative real-time polymerase chain reaction (qRT-PCR) according to a protocol by Cawthon (17). This method measures relative telomere length by determining the ratio of telomere repeat copy number to single copy gene 36B4 in individual samples relative to a standard pooled DNA with a 7500 FAST real-time PCR system (Applied Biosystems, CA). Briefly, the PCR reaction (20 μL) for the telomere or 36B4 amplification consisted of 2X SYBR Green Master Mix (Thermo Scientific), 250 nmol/L each telomere or 36B4 specific primers, and 4 μL purified cfDNA sample. The thermal cycling conditions for both telomere and 36B4 were 95°C for 10 min followed by 40 cycles of 95°C for 15 s, 58°C for 30 s, and 72°C for 59 s with signal collection. The primer sequences were as follows: forward telomere primer (Tel-1), 5′-CGGTTTGTTTGGGTTTGGGTTTGGGTTTGGGTTTGGGTT-3′; reverse telomere primer (Tel-2), 5′-GGCTTGCCTTACCCTTACCCTTACCCTTACCCTTACCCT-3′; forward human 36B4 primer, 5′-CAGCAAGTGGGAAGGTGTAATCC-3′; reverse human 36B4 primer, 5′-CCCATTCTATCATCAACGGGTACAA-3′.

For quality control and calibration of PCR efficiency, the same standard DNA extracted from pooled serum samples were included on each plate with all samples assayed in duplicate. Melting curve analysis was performed after every reaction to verify specificity and identity of the PCR products. The standard DNA was diluted using 3-fold increment per dilution to produce a seven-point standard curve, ranging from 0.05 to 40 ng/μL template DNA. The standard curves for amplifications of telomere and 36B4 were constructed every 10 plates before the detection of samples, with all the *R*^2^ values were ≥0.99. The samples with low concentration of cfDNA (Ct value of 36B4 >34) were deleted for out of the linearity of the standard curve. The intra-assay coefficient of variation for telomere/36B4 ratio was <8.0%. The average coefficients of variation were 3.1% for telomere reaction and 2% for 36B4 reaction, respectively. When the coefficient of variation was higher than 15%, the measurement was repeated. The paired samples were analyzed on the same plate by one technician with group information blind.

### *H. pylori* Antibody Assay

As described previously, *H. pylori* antibody assays were used to determine infection status in 1994, 1996, 1997, and 1999 (18). In brief, serum levels of anti-*H. pylori* IgG and IgA were measured separately in duplicate with enzyme-linked immunosorbent assay procedures. An individual was defined to be positive for *H. pylori* infection if the mean optical density for either IgG or IgA was 1.0. Quality control samples were assayed at Vanderbilt University, Nashville, TN.

### Statistical Analysis

For the baseline data analysis, the distributions of general characteristics between GC subjects and controls were compared by the chi-square tests for categorical variables and paired *t*-test for age. Telomere lengths were compared between subjects who progressed to GC and their paired controls by Wilcoxon rank-sum test. The odds ratio (OR) and corresponding 95% confidence interval (CI) for the association between baseline telomere length and GC risk were calculated by conditional logistic regression adjusting for age, *H. pylori* infection and gastric lesion status. Within different categories of baseline gastric lesion, the associations between baseline telomere length and GC risk were assessed by unconditional logistic regression adjusting for age and *H. pylori* infection.

Using multiple measurements of samples from all collection time-points, linear mixed models were applied to examine the associations between cfDNA telomere length and potential influencing factors by the regression coefficients and 95%CIs. The association between cfDNA telomere length in multiple time-points and the risk of GC was further evaluated by generalized linear mixed model. The changes of telomere length before and after interventions were compared between active and placebo groups by Wilcoxon rank-sum test. To compare the temporal trends of cfDNA telomere length, all the pre-diagnostic samples were sub-grouped according to the years before GC-diagnosis and compared with their corresponding controls by linear regression adjusting age. The annual attrition rate of telomere length in a specific time span for each subject was calculated by dividing the telomere length difference between two consecutively collected serum samples by interval years and baseline telomere length. Therefore, we had 3 average annual telomere attrition rates for subjects with 4 serum samples, and 2 average annual telomere attrition rates for subjects with 3 samples. Differences of average annual attrition rates between GC and control groups were compared by Wilcoxon rank-sum tests.

All statistical analyses were performed using the SAS software version 9.2 (SAS Institute, Cary, NC). All statistical tests were two-sided, and *P* < 0.05 was considered as statistically significant.

## Results

### Selected Characteristics of the Subjects in GC and Control Groups

The baseline characteristics and the intervention assignment of the subjects in GC and control groups were shown in Table 1. Compared with controls, GC group had a significantly higher proportion of *H. pylori* infection (81.4 vs. 58.1%, *P* < 0.001), and more advanced gastric lesions such as IM or DYS (46.5, 40.7 vs. 31.4, 13.9%, *P* < 0.001). No significant differences were observed between GC cases and controls in age, sex, smoking, alcohol intake and any of the three interventions.

**Table 1:** Selected characteristics of subjects in GC and control groups.

**Variables**	**Total**	**GC**	**Control**	***P***
	**(*n* = 172)**	**(*n* = 86)**	**(*n* = 86)**	
Baseline characteristics				
Age (years, mean ± SD)	50.8 ± 9.6	51.9 ± 9.7	49.8 ± 9.5	>0.999^a^
Sex (%)				>0.999^b^
Female	42 (24.4)	21 (24.4)	21 (24.4)	
Male	130 (75.6)	65 (75.6)	65 (75.6)	
Smoking (%)				0.519^b^
Never	58 (33.7)	27 (31.4)	31 (36.1)	
Ever	114 (66.3)	59 (68.6)	55 (63.9)	
Drinking (%)				**>**0.999^b^
Never	60 (34.9)	30 (34.9)	30 (34.9)	
Ever	112 (65.1)	56 (65.1)	56 (65.1)	
*H. pylori* infection (%)				<0.001^b^
Negative	52 (30.2)	16 (18.6)	36 (41.9)	
Positive	120 (69.8)	70 (81.4)	50 (58.1)	
Gastric lesions (%)				<0.001^b^
Normal/SG/CAG	57 (33.1)	10 (11.6)	47 (54.7)	
IM	67 (39.0)	40 (46.5)	27 (31.4)	
DYS	47 (27.3)	35 (40.7)	12 (13.9)	
Missing	1 (0.6)	1(1.2)	–	
Intervention treatments assigned in 1995				
Anti-*H. pylori* treatment^c^				0.339^b^
No	59 (49.2)	37 (52.9)	22 (44.0)	
Yes	61 (50.8)	33 (47.1)	28 (56.0)	
Garlic supplement				0.360^b^
No	88 (51.2)	47 (54.7)	41 (47.7)	
Yes	84 (48.8)	39 (45.3)	45 (52.3)	
Vitamin supplement				**>**0.999*^b^*
No	90 (52.3)	45 (52.3)	45 (52.3)	
Yes	82 (47.7)	41 (47.7)	41 (47.7)	

a *Equivalence t-test*.

b *Pearson's Chi-square test without missing values*.

c *Anti-H. pylori treatment and corresponding placebo was only assigned to 120 H. pylori positive subjects*.

*CAG, chronic atrophic gastritis; DYS, dysplasia; GC, gastric cancer; H. pylori, Helicobacter pylori; IM, intestinal metaplasia; SD, standard deviation; SG, superficial gastritis*.

### Relationships Between Epidemiologic Parameters and Telomere Length of cfDNA

With a total of 276 pairs of serum samples collected at baseline or three follow-up time-points from the 86 pairs of GC and control subjects, we firstly evaluated the associations between telomere length of cfDNA and age or other potential influencing factors (Table 2). Linear mixed model found that aging was a significant risk factor for telomere shortening (regression coefficient = −0.009, *P* < 0.001 per 1 year older), after adjusting with other factors in multivariate model. No significant associations were found between cfDNA telomere length in serum and sex, smoking, alcohol drinking or *H. pylori* infection status (all *P* > 0.05).

**Table 2:** Relationships between epidemiologic parameters and telomere length of cfDNA from baseline and follow-up time-points.

**Variables**	**Unadjusted model^a^**		**Adjusted model^b^**	
	**Regression coefficient (95%CI)**	***P***	**Regression coefficient (95%CI)**	***P***
Age	−0.009 (−0.012, −0.006)	<0.001	−0.009 (−0.012, −0.005)	<0.001
Sex				
(Female = 0, Male = 1)	−0.056 (−0.132, 0.020)	0.150	−0.050 (−0.169, 0.069)	0.409
Smoking				
(Never = 0, Ever = 1)	−0.079 (−0.147, −0.011)	0.024	−0.040 (−0.147, 0.066)	0.455
Drinking				
(Never = 0, Ever = 1)	−0.008 (−0.076, 0.061)	0.824	0.042 (−0.049, 0.133)	0.366
*H. pylori* infection				
(Negative = 0, Positive = 1)	−0.025 (−0.096, 0.046)	0.490	−0.044 (−0.114, 0.027)	0.223

a *Univariate linear mixed model*.

b *Multivariate linear mixed model adjusting for age, sex, smoking, alcohol intake, and H. pylori infection status*.

*CI, confidence interval; H. pylori, Helicobacter pylori*.

### Effects of Interventions on Telomere Length of cfDNA

To evaluate the effects of the three interventions on serum cfDNA telomere length, we calculated the change of telomere length in 86 *H. pylori*-positive subjects with serum samples from 1994 and 1996 for the comparison between anti-*H. pylori* and placebo treatments, and in 104 subjects with serum samples from 1994 and 1999 for the comparison between long-term supplementation of garlic or vitamin and placebo treatments. No significant differences in change of telomere length were found between any active treatments and corresponding placebo groups, with *P*-values as 0.587 for anti-*H. pylori* treatment, 0.363 for garlic supplementation and 0.457 for vitamin supplementation (Table 3).

**Table 3:** Changes of cfDNA telomere length after intervention.

**Intervention treatments assigned in 1995**	**Total**		
	***n***	**Changes of telomere length median (interquartile range)**	***P*^e^**
			
Anti-*H. pylori* treatment^a^^,^ ^c^			0.587
No	41	0.13 (-0.34, 0.69)	
Yes	45	0.42 (-0.12, 0.83)	
Garlic supplement^b^^,^ ^d^			0.363
No	54	1.23 (0.75, 1.67)	
Yes	50	1.07 (0.71, 1.47)	
Vitamin supplement^b^^,^ ^d^			0.457
No	56	1.21 (0.71, 1.71)	
Yes	48	1.11 (0.86, 1.36)	

a *For anti-H. pylori treatment, the change of telomere length was defined as the difference of serum cfDNA telomere length between 1994 and 1996*.

b *For garlic supplement and vitamin supplement, the change of telomere length was defined as the difference of serum cfDNA telomere length between 1994 and 1999*.

c *A total of 86 subjects who received anti-H. pylori or placebo treatment in 1995 and possessed serum samples both from 1994 and 1996 were analyzed*.

d *A total of 104 subjects who received supplementation of garlic or vitamin or corresponding placebos and possessed serum samples both from 1994 and 1999 were analyzed*.

e *Wilcoxon rank-sum test*.

### The Associations Between Baseline cfDNA Telomere Length and Risks of GC or Precancerous Lesions

Among 86 pairs of GC and control subjects, 73 pairs possessed serum samples at baseline in 1994. The baseline cfDNA telomere length median was shorter in those who progressed to GC during the follow-up period than in controls, *P* < 0.001. Shortened cfDNA telomere length at baseline was associated with increased subsequent GC risk (OR = 9.17, 95%CI: 2.72, 31.25, *P* < 0.001, for 1 unit shortening), after adjusting with age, *H. pylori* infection and gastric lesions (Table 4).

**Table 4:** Associations between telomere length of cfDNA and GC risks.

	**GC**		**Control**			
	***n***	**cfDNA telomere length median (interquartile range)**	***n***	**cfDNA telomere length median (interquartile range)**	**OR (95%CI)**	***P***
Total subjects	73	1.37 (1.08–1.74)	73	2.17 (1.76–2.51)	9.17^b^ (2.72–31.25)	<0.001^b^
Baseline gastric lesions^a^						
Mild gastric lesions	10	1.36 (1.08–1.86)	39	2.18 (1.76–2.48)	6.99^c^ (1.63, 30.30)	0.009^c^
IM	35	1.26 (1.08–1.74)	23	2.16 (1.60–2.79)	6.06^c^ (1.89, 19.61)	0.002^c^
DYS	27	1.52 (1.09–1.87)	11	2.19 (1.81–2.65)	15.63^c^ (1.91, 125.00)	0.010^c^

a *One subjects was excluded for missing pathology diagnosis at baseline*.

b *Conditional logistic regression analysis for the OR per one unit decrease of telomere length, adjusting for age, H. pylori infection and gastric lesions*.

c *Unconditional logistic regression analysis for the OR per one unit decrease of telomere length, adjusting for age, H. pylori infection. cfDNA, cell-free DNA; CI, confidence interval; DYS, dysplasia; GC, gastric cancer; IM, intestinal metaplasia; OR, odds ratio*.

In addition to the risk of GC, telomere lengths were also analyzed among different baseline gastric lesions. Statistical difference was found in telomere length medians (interquartile range, IQR) among various gastric lesion groups [mild gastric lesions (no more than chronic atrophy gastritis): 2.12 (IQR, 1.35–2.40); IM: 1.55 (IQR, 1.17–2.16); DYS: 1.71 (IQR, 1.22–1.99), *P* = 0.038]. While after adjusting with age and *H. pylori* infection, unconditional logistic regression showed no significant associations between shortened cfDNA telomere and IM (OR = 1.56, 95%CI: 0.88–2.75 for 1 unit shorting) or DYS (OR = 1.62, 95%CI: 0.86–3.07 for 1 unit shorting) with mild gastric lesions group as reference.

The risks of subsequent GC progression were further investigated in different categories of baseline gastric lesion. Telomere length medians were significantly shorter in GC than in control group for each baseline lesion category. Significantly higher risks of GC progression were also found by multivariate analysis with shortened cfDNA telomere length; ORs per 1 unit shortening were 6.99 (95% CI: 1.63–30.30, *P* = 0.009) for mild gastric lesions, 6.06 (95% CI: 1.89, 19.61, *P* = 0.002) for IM and 15.63 (95% CI: 1.91, 125.00, *P* = 0.010) for DYS (Table 3).

### The Association Between cfDNA Telomere Length and the Risk of Subsequent GC Based on All Serums From Four Collection Time-Points

Considering the significant association between baseline cfDNA telomere length and GC risk, we further evaluated the association using 276 pairs of serum samples from all four collection time-points by generalized linear mixed model. After adjusting with age, smoking, drinking, *H. pylori* status, baseline gastric lesions and calendar year of serum collection, shortened cfDNA telomere length was significantly associated with higher risk of GC progression (OR = 7.37, 95%CI: 2.06, 26.32, *P* = 0.002, for 1 unit shortening).

### Temporal Trend of cfDNA Telomere Length in GC Development

To investigate the temporal trend of cfDNA telomere length in the process of gastric carcinogenesis, 276 pairs of serum samples from GC subjects and corresponding matching controls were assigned into sub-groups according to the interval years between sample collection and clinical diagnosis of GC (Figure 1). Compared to their corresponding controls, GC subjects showed shorter age-adjusted telomere lengths for most interval year sub-groups from 4 to ≥14 years (*P* < 0.05, except for the sub-group of 8 or 10 years). From 1 to 3 years before GC-diagnosis, no significant differences were found between progress-to-GC group and matching control group (*P*-values as 0.148, 0.235, and 0.115, respectively).

### The Association Between Annual Telomere Length Attrition Rates and the Risk of GC

**Figure 2:**
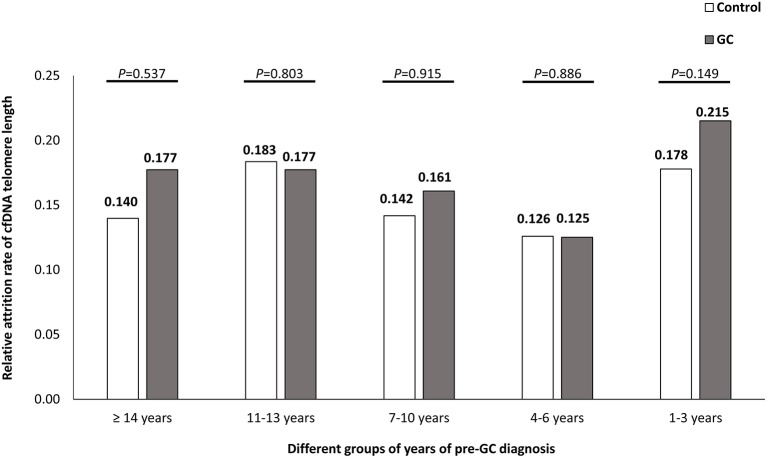
The temporal trends of relative annual telomere attrition rates in GC and control groups. The years before GC-diagnosis of pre-diagnostic samples from GC subjects were classified into five groups as ≥14 years, 11–13 years, 7–10 years, 4–6 years, and 1–3 years, respectively. The annual attrition rates between GC and control groups were compared by Wilcoxon rank-sum tests.

The medians of annual attrition rate were 0.165/year (IQR, −0.001 to 0.277/year) in GC group and 0.157/year (IQR, 0.004–0.251/year) in control group, respectively. No significant difference was found between the two groups, *P* = 0.656. We further divided the interval years between serum collection and GC-diagnosis into five categories (≥14 years, 11–13 years, 7–10 years, 4–6 years, and 1–3 years). The annual attrition rate medians of telomere length in GC and control groups showed no significant difference in each category (Figure 2).

## Discussion

Based on a long-term follow-up study in a high-risk population of GC, we found that subjects with shortened cfDNA telomere lengths were associated with a higher risk of GC progression. Moreover, shortened cfDNA telomere length can be detected more than 3 years ahead of GC diagnosis. To our best knowledge, this is the first population-based prospective study to dynamically explore telomere length of serum cfDNA in the process of GC carcinogenesis.

Although associations between telomere length and cancer risk or prognosis have been extensively investigated in tumor tissues in the past decades (19–21), telomere in peripheral blood leukocytes is attracting more attentions for its non-invasion and accessibility in the recent studies (22). However, inconsistent results were found in most of the case-control studies with positive (longer telomere with higher cancer risk), negative (shorter telomere with higher cancer risk), or even U-shape associations (9, 22). A previous longitudinal study based on American Normative Aging Cohort suggested longer blood leukocyte DNA telomere only occurred at 3–4 years ahead of cancer incidence (23). While, the main cancer types in this cohort were prostate and skin tumors, which may limit the extension of the result to GC. Our study demonstrated no significant association between the telomere length of blood leukocyte DNA and GC risk (data not shown). The divergence of these studies suggested that the homogeneous hematopoietic blood leukocyte DNA telomere length may not be a sensitive cancer predictor, and alternations are needed, such as cfDNA from serum.

The origins of cfDNA are not totally clear until now. Increasing evidences suggested that it might be passively released by apoptotic and necrotic cells as DNA fragments (24) or actively excreted from distant tumor cells for signal transmission (25). The higher amount of cfDNA was reported from serum compared to plasma (26). Our study found increased concentration of cfDNA in serum within 5 years ahead of clinical diagnosis of GC (data not shown). These results suggested the less degraded serum cfDNA as a better biomarker to synthetically reflect the alterations from infection, inflammation, and carcinogenesis in the whole body. Although the fragmented status of cfDNA may limit the accuracy of quantitative PCR, our well-designed study with matched serum pairs from the same follow-up time-point can relatively show the telomere length difference between GC cases and controls. The significantly shortened cfDNA telomere in the pre-diagnostic serums of GC subjects from baseline and different follow-up time-points further confirmed it as an early event in gastric carcinogenesis.

Precancerous gastric lesions were previously reported to be associated with shorter telomere (6). A recent prospective genomic and epigenetic profiling study revealed that telomeres were initially reduced in high risk IMs (who developed to GC), but subsequently restored during GC progression (27) for the activation of telomere maintenance mechanisms (4, 28). In the current study, we also found shortened baseline cfDNA telomeres especially in IM subjects, while the telomeres were restored in DYS. When stratified by baseline lesion categories, the higher risk of GC progression for subjects with shortened telomere in each category further confirmed that aberrant alteration of cfDNA telomere may be associated with gastric carcinogenesis from early stage of precancerous lesions. While further prospective validation in larger number of precancerous gastric lesion subjects is still needed.

Although shorter telomeres in high risk IMs were reported previously (27), only 6 IMs progressing to DYS or GC in that cohort limited the generalization of the results. Our prospective study dynamically explored cfDNA telomere length changes in 86 GCs identified in 16-year follow-up period and found significantly shortened telomeres from 4 years to more than 14 years ahead of GC-diagnosis. Our results may reflect the comprehensive effects during the long-term carcinogenesis process, such as more severe inflammation and precancerous lesions in those who progressed to GC subsequently. In addition, genetic variations regulating individual telomere length may also be involved in the susceptibility of GC more than 10 years before clinical diagnosis in the present study (29). Although no serums were detected after GC-diagnosis in our study, non-significant differences between two groups from 1 to 3 years ahead of GC diagnosis may also support the restoration of telomere after cancer incidence.

With 2–4 serum samples collected for each subject in long-term follow-up period, our study had a unique opportunity to calculate the average annual telomere attrition rates. For the first time, we described the alteration of telomere attrition rates from 1 to more than 14 years before GC diagnosis, although no significant difference was found between GC and control groups.

The mechanism of telomere shortening has been well-explained by incomplete synthesis of chromosomal ends with cell division (30), which can be modified by physiological and pathological factors, such as aging (31), inflammation (32), and carcinogenesis (4). Consistent with previous studies (33, 34), our study found shorter cfDNA telomeres in older subjects and continuous telomere shortening in temporal trend analysis in control group, which may be caused by aging. Although infection of *H. pylori*, an important risk factor of GC, was reported to be associated with shorter telomere length in gastric mucosa (35), we did not found a remarkable relationship between *H. pylori* infection status and cfDNA telomere length at baseline. Based on the large intervention trial, our study is further capable of analyzing the effects of anti-*H. pylori* treatment and supplementation of garlic or vitamin on cfDNA telomere length. With the similar distributions of the three intervention arms between GC and control groups, no effective influence on telomere length alteration was found 1 year after 14-day anti-*H. pylori* treatment and after 4-year supplementation of garlic or vitamin, although further long-term effects still need longer follow-up with more bio-sample collection in the future.

The major strength of our study lies in the prospective design, which enabled us to estimate temporal associations between dynamic telomere alterations and GC risk with multiple pre-diagnostic serum samples from GC subjects and corresponding controls based on the same cohort. A limitation of our study is the relative small number of GC subjects. Only 86 GC cases were enrolled with the multiple time point pre-diagnostic serum samples. Individual matched controls were also strictly selected from the same 16-year prospective cohort in Linqu County. Multicenter confirmation with larger sample size is still needed for the extrapolation of the present results. In addition, the mechanism underlying the significant association between cfDNA telomere length and GC risk still require future studies.

In conclusion, our population-based study provided evidence for the first time that aberrant alterations of cfDNA telomere length may happen early in the process of GC development. The dynamic observation of telomere shortening may provide us clues for further etiological study on gastric carcinogenesis and may serve as a potential non-invasive marker for high-risk population screening and monitoring.

## Data Availability Statement

The raw data supporting the conclusions of this manuscript will be made available by the authors, without undue reservation, to any qualified researcher.

## Ethics Statement

This study was carried out in accordance with the recommendations of the Institutional Review Board of Peking University Cancer Hospital with written informed consent from all subjects. All subjects gave written informed consent in accordance with the Declaration of Helsinki. The protocol was approved by the Institutional Review Board of Peking University Cancer Hospital.

## Author Contributions

W-CY and K-FP: study design and manuscript proofing. D-JD: experimental design. LZ, J-LM, TZ, Z-XL, W-DL, and W-QL: data collection. YS and YZ: experimental operation, data analyses, and manuscript writing. All authors: results interpretations.

### Conflict of Interest

The authors declare that the research was conducted in the absence of any commercial or financial relationships that could be construed as a potential conflict of interest.
